# Osmotic pressure induced tensile forces in tendon collagen

**DOI:** 10.1038/ncomms6942

**Published:** 2015-01-22

**Authors:** Admir Masic, Luca Bertinetti, Roman Schuetz, Shu-Wei Chang, Till Hartmut Metzger, Markus J. Buehler, Peter Fratzl

**Affiliations:** 1 Department of Biomaterials, Max Planck Institute for Colloids and Interfaces, Research Campus Golm, 14424 Potsdam, Germany; 2 Laboratory for Atomistic and Molecular Mechanics, Department of Civil and Environmental Engineering, MIT, Cambridge, Massachusetts 02139, USA

## Abstract

Water is an important component of collagen in tendons, but its role for the function of this load-carrying protein structure is poorly understood. Here we use a combination of multi-scale experimentation and computation to show that water is an integral part of the collagen molecule, which changes conformation upon water removal. The consequence is a shortening of the molecule that translates into tensile stresses in the range of several to almost 100 MPa, largely surpassing those of about 0.3 MPa generated by contractile muscles. Although a complete drying of collagen would be relevant for technical applications, such as the fabrication of leather or parchment, stresses comparable to muscle contraction already occur at small osmotic pressures common in biological environments. We suggest, therefore, that water-generated tensile stresses may play a role in living collagen-based materials such as tendon or bone.

## Abstract (web summary)

Water is an important component of collagen in tendons, bone and extracellular matrix, but its role in the mechanical function of protein is poorly understood. Here, the authors study the effects of osmotic pressure on contraction in collagen, suggesting that collagen could function as a mechanical actuator.

## Graphical abstract

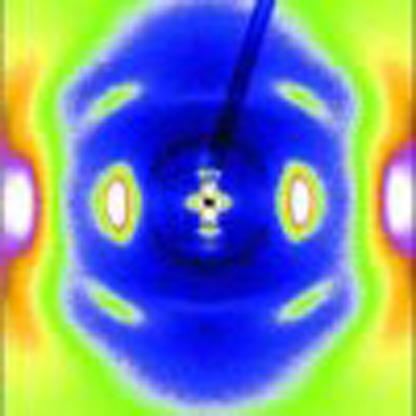

Tendons are known to transmit force between muscles and bone. They are composed of collagen and proteoglycans and—not least—of 62 wt% water^1^. Given that tendons work in a hydrated environment, the role of water generally does not attract wide interest, except when dehydration is considered explicitly, as in the fabrication of leather or parchment^2^,^3^. It is well-known that the mechanical properties of collagen change drastically upon dehydration, but the origin of this effect is poorly understood. Even less clear is whether there is a direct role of water for the function of collagen in the native (hydrated) state^4^,^5^,^6^,^7^. Collagen is known to be built in a hierarchical fashion with triple-helical collagen molecules being assembled in a staggered fashion into fibrils, which are embedded in a proteoglycan-rich matrix and further assembled into fibres and fascicles^8^,^9^,^10^,^11^. The axial molecular staggering (*D*) in fibrils is typically 67 nm in wet type I collagen and reduces to about 64 nm when dry^5^. Laterally, the molecules are packed with a spacing in the order of 1.5 nm, which reduces to 1.1 nm when water is removed^12^,^13^.

When loaded in tension, collagen fibrils deform in a complex scale-dependent fashion where fibrils stretch more than the molecules and the whole tendon more than fibrils^14^,^15^,^16^,^17^. This has been explained through a side-by-side gliding of molecules and fibrils, enabled by the multi-scale structure of the tendon, based on *in situ* experimentation with synchrotron radiation^15^ and on multi-scale computational modelling^18^. Polarized Raman imaging has been shown to complement the picture with information about molecular conformation and orientation under load^19^.

Here we combine all of these experimental and theoretical approaches to unravel the role of water in type I collagen structure and mechanical behaviour. The approach consists of measuring the hydration-dependent molecular features of collagen, both with and without applied load, using X-ray diffraction and Raman spectroscopy. To allow the quantitative control of the osmotic pressure acting on the collagen fibre, we studied it in a chamber with controlled relative humidity (RH) that was then converted to osmotic pressure *Π*, using the well-known relation *Π=RT/v*_m_*·ln(*RH), where *R* is the gas constant, *T* the temperature and *v*_m_ the molar volume of water. Changes in fibril structure are assessed by small-angle X-ray scattering (SAXS) and diffraction, techniques used over the past few decades to study the molecular and supramolecular structure of collagen^20^,^21^. Complementary information on collagen conformation is obtained through the targeted re-analysis of previously described molecular dynamics (MD) modelling results^22^. We find major water-induced conformational changes of the collagen molecule, which are very inhomogeneous along its length. We also show that the resulting shortening is capable of producing tensile stresses, which—depending on osmotic pressure—may get up to 100 MPa, much larger than that generated by muscles (peak stresses in the order of 0.3 MPa (ref. 23)).

## Results

### Structural characterization

**Figure 1:**
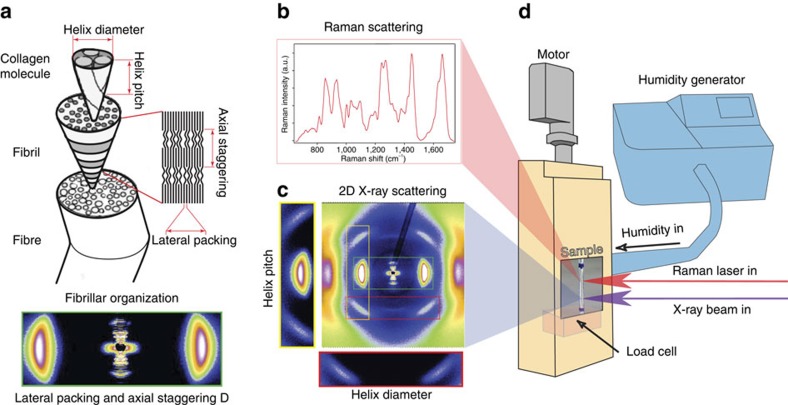
Experimental setups for *in situ* analysis of hierarchical structure of tendon. The hierarchical structure of tendon (**a**) requires a range of experimental techniques (**b**,**c**) to provide quantitative *in situ* (**d**) information at different length scales. Tensile stage with humidity and temperature control (**d**) used either with Raman scattering (**b**) or synchrotron X-ray diffraction (**c**). Raman scattering shows peaks characteristic for collagen protein backbone and its conformation. The two-dimensional X-ray pattern (**c**) provides information on the pitch and the diameter of the collagen triple-helix as well as on the axial staggering and lateral spacing of these molecules.

In order to assess tendon collagen structure at various length scales, we employ several *in situ* methods under controlled environmental conditions (temperature and humidity, Fig. 1a). In particular, tendon is composed of fibrils with a diameter typically on the order of a hundred or a few hundred nanometers. Within these fibrils, triple helical collagen molecules are staggered with an axial D-period of 67 nm (ref. 24). Given that the length of the molecule is not an integer multiple of the D-period, the staggering leads to a succession of gap and overlap zones along the fibril length, where the gap zones have a lower molecular density (see Fig. 1a). The effects of hydration on collagen backbone conformation in rat tail tendon (RTT) are monitored by Raman scattering (Fig. 1b), whereas changes in triple helix parameters (radius, pitch and lateral spacing) are measured using synchrotron X-ray diffraction (Fig. 1c). SAXS reveals hydration-dependent changes of the collagen staggering period (*D*) and of the relative lengths of gap and overlap in the fibrils, whereas macroscopic deformation and forces generated are measured using a custom built micromechanical tensile tester (Fig. 1d). By means of full atomistic MD, we have gained further insight into molecular level changes of collagen structure with and without loading, and with varied amounts of water molecules present.

### RTT contraction and force generation upon dehydration

**Figure 2:**
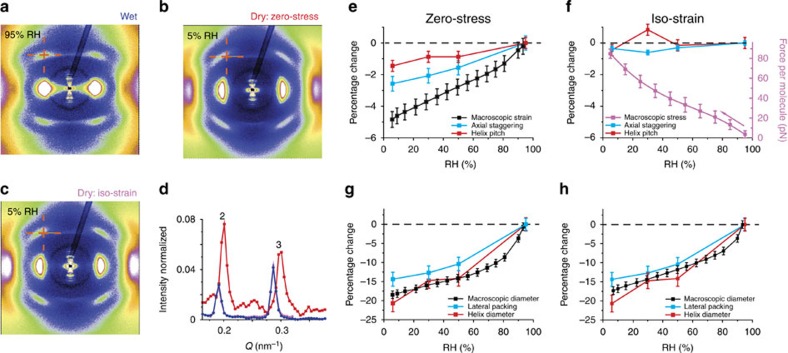
Multilevel analysis of the mechanical behaviour of rat tail tendon. (**a**) 2D X-ray scattering in the wet state and after drying (**b**) without load (zero-stress) or (**c**) at constant length (iso-strain). The cross in **a**–**c** indicates the position of the scattering maximum of the collagen triple-helix in the wet state (**a**). Note the shift and deformation of the triple helix feature during drying. (**d**) Second and third order reflection from axial staggering, normalized to the same intensity in the first order. The curves for the hydrated and the iso-strain case overlap (blue and violet), whereas the peak position and intensities are changed after drying with no load (red). (**e**–**h**) Humidity dependence of structural parameters at different length scales represented as changes relative to the fully hydrated state. (**e**,**f**) Dimensions in axial direction are shown, the macroscopic length of the tendon (black), the axial staggering period of the molecules (blue) and the average helix pitch inside the molecule (red). In the case of drying at constant length, (**f**) the force per unit molecule is also reported (violet curve). (**g**,**h**) Dimensions in lateral direction are shown, the macroscopic diameter (black), the lateral spacing between molecules (blue) and the diameter of the triple-helix (red). (**e**,**g**) The dehydration without load and (**f**,**h**) at constant length are presented. The forces generated on the order of tens of pN are sufficiently large to yield unfolding of collagen molecules, as the force exceeds the critical range for purely entropic forces and induces unwinding mechanisms, which was estimated to be around 20–30 pN (ref. 25).

Figure 2a–c shows characteristic diffraction patterns of wet RTT (Fig. 2a) and after drying either without load (Fig. 2b) or at constant length (Fig. 2c). Shifts of the scattering maximum due to the collagen triple-helix (as indicated by red crosses in Fig. 2a–c) can clearly be observed. By means of these patterns, quantitative structural molecular and supramolecular parameters in both axial and lateral directions were assessed as functions of humidity and macroscopic loading conditions (Fig. 2e–h, see also Supplementary Figs 1–4). In Fig. 2e,f, changes of fibril length (as measured by the axial staggering period of the collagen, blue) and of the molecule length (as measured by the average helix pitch, red) are plotted together with the macroscopic length of the tendon for a zero-stress experiment (black). In addition, this figure also shows the stress (expressed as force per unit collagen molecule, violet curve) generated in a dehydration experiment at constant length. The forces generated on the order of tens of pN are sufficiently large to yield conformational changes of collagen molecular structure, as the force exceeds the critical range for purely entropic forces and induces unwinding mechanisms, estimated to be around 20–30 pN (ref. 25). Unfolding under tensile stress is found for other components of the extracellular matrix, such as fibronectin^26^, where the conformational change was shown to be associated with a different biochemical signalling. Although tendon shrinks by up to ~5% at the macroscopic level (Fig. 2e, black), fibrils contract only 2.5% as measured by changes in the axial staggering. The extent of change in axial parameters is even less at the level of the triple helix (helix pitch, Fig. 2e, red) where only ~1.3% contraction was found. Interestingly, in the experiment at constant length (Fig. 2f), both fibrillar and molecular level parameters, as well as the gap to overlap ratio (reflected in the ratio of 2nd to 3rd order peaks, Fig. 2d), do not significantly change. The intensity changes are only found when the tendon is dried without applied load (red curve, Fig. 2d) indicating that dehydration alters the gap/overlap ratio at the fibrillar level only if the tendon is left to strain axially. Hindering the strain by clamping the fibre is, however, accompanied by a remarkable stress generation that reaches 106±16 pN per molecule at 5% RH (Fig. 2f, violet).

In the lateral direction (Fig. 2g,h), very similar percentage changes (around 20%) were found in both experimental conditions (zero-stress and iso-strain) for all investigated levels of structure (macroscopic fibre diameter (black), the lateral spacing between molecules (blue) and the diameter of the triple-helix (red)). Latter results are in agreement with data reported in the literature where the packing of the collagen molecules at fibrillar level^27^ and thinning of the fibre at macroscopic scale were observed. However, the average changes in helix diameter (up to 25% for completely dry tendon in both experimental configurations) are surprising, as such an increase on the helix radius at constant chain length would imply a dramatic decrease in the triple helix length.

### Water as an integral part of the collagen molecule

**Figure 3:**
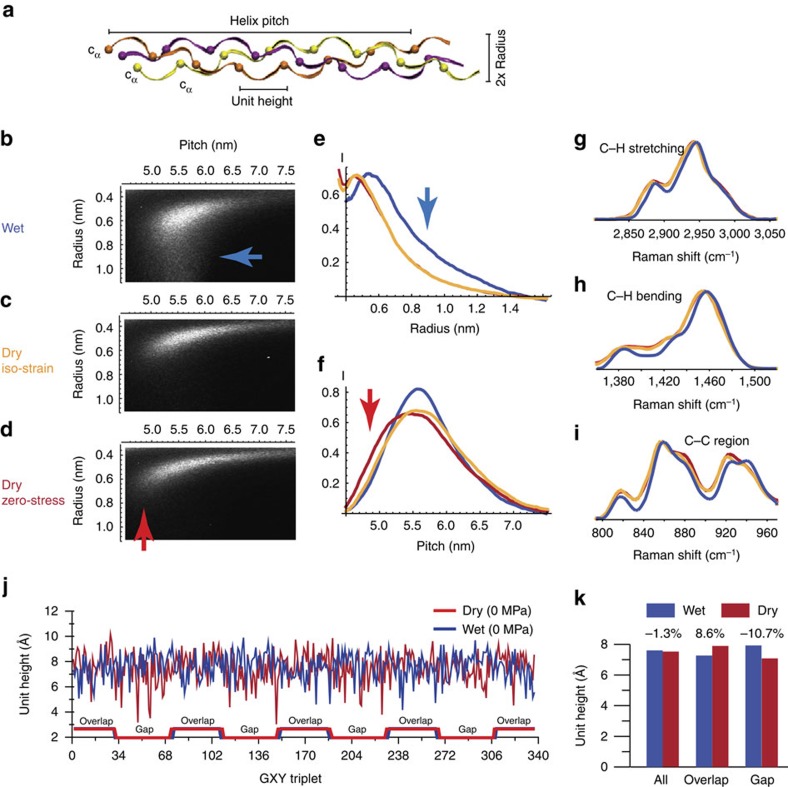
Conformational changes of the collagen triple helix during dehydration. (**a**) Cartoon of the collagen triple helix defining pitch, radius and unit height. 2D distributions of intensities relative to helix radii and helix pitches calculated from X-ray diffraction data (**b**–**d**) are plotted together with the marginal distributions obtained by projecting the 2D distributions on the marginal variable axes (**e** and **f**). Measured projections refer to the wet (blue curves) and two dry states (red and green curves for zero-stress and iso-strain, respectively). Raman spectra of CH stretching (**g**), CH bending (**h**) and CC stretching (**i**) vibrations for wet (blue curves) and dry (red and green curves for zero-stress and iso-strain respectively) collagen, indicating conformational changes of the collagen backbone induced by dehydration. (**j**) Analysis of unit height changes in a full atomistic collagen microfibril model in the wet and dry states with no load applied^22^. (**k**) Average changes of the unit height in the gap and the overlap zones obtained from the full atomistic collagen microfibril model.

A better understanding of the changes that the collagen molecule undergoes when surrounding water is removed can be obtained by re-drawing the X-ray scattering patterns to extract the helix parameters (as defined in Fig. 3a) at different hydration conditions (details on the data treatment can be found in Supplementary Fig. 1 and Supplementary Methods section. The equations reported in Supplementary Fig. 1 and used to calculate the helix parameters from the scattering patterns are derived following the comprehensive treatment by Okuyama^28^). In Fig. 3, the distribution of helix pitches and helix radii for the collagen fibre (Fig. 3b–d) together with the marginal distributions (Fig. 3e,f) obtained by projecting the two-dimensional (2D) distributions on the marginal variable axes are reported. Rigorously, these projections are influenced by size and imperfections of the domains of constants helix pitch (finite-size broadening). As the data do not allow us to deconvolute these effects, we can only give a qualitative interpretation of some of the observations. The variations of the raise per residue within the collagen molecule were very nicely modelled by Cameron *et al*.^29^ and results are consistent with our findings, given the limitations of our measurements: the Cameron model predicts a 25% variation in the raise per residue, while the width of the peak corresponds to 40%. The fact that the measured value is larger probably reflects the additional finite-size broadening. Moreover, water molecules are bound to the triple-helical collagen molecules in the hydrated state and ‘decorate’ it in such a way that water contributes to the helix diffraction spot. Hence, the radius of the helix may appear wider, that is, with a larger radius (blue arrow in Fig. 3b). Furthermore, the distribution of helix pitches widens with de-hydration, suggesting that the structure of the collagen triple helix is affected by the presence of water. In particular, water molecules seem to stabilize the collagen triple helix, as the pitch distribution is more homogeneous in the wet state. Although these structural changes are substantial, the average pitch of the helix decreases only by 1.3% from the wet to the dry state, as shown in Fig. 2e. In an experiment where the tendon is not allowed to strain in axial direction, the average helix pitch remains constant and the pitch distribution is broadened almost symmetrically.

### Local conformational changes of the collagen backbone

The effects of water on the collagen molecular structure can also be observed using *in situ* Raman spectroscopy. Significant changes in the band shape and position of the amide bands (see Supplementary Fig. 5) as well as the C–H and C–C vibrational modes (Fig. 3g–i) are detected while dehydrating. These effects, as expected, are very pronounced for the amide bands, as these are very sensitive to the electron density change of the collagen backbone induced by the formation/disruption of H-bonds with water molecules (see Supplementary Fig. 5). Although the analysis of the polarized Raman spectra of amide bands could be carried out following the methodologies developed in some recent papers^19^,^30^, this treatment is complicated by the possible simultaneous occurrence of changes in electronic structure and in molecular orientation. Moreover, the broadening observed for the C–H and the C–C stretching as well as the C–H bending modes indicates that the local structure around these molecular moieties becomes more heterogeneous when water is removed from the surrounding of the triple helix, confirming our X-ray and previous nuclear magnetic resonance findings^31^. Also, as these vibrations are almost unaffected by electronic effects, the shifts in the band position confirm that the triple helix backbone undergoes extensive conformational changes.

To explore the molecular level transformations in greater details, we analyse the data obtained previously in MD simulations of the collagen fibril, which includes N-, C-telopetide domains and full type I collagen sequence in hydrated and dehydrated conditions^22^. This atomistic collagen microfibril model provides further insights into the structural changes induced by water and allows us to extract atomic-level structural information. Comparing the unit heights of the wet and dry triple helix along the polypeptide chain, the numerical model shows that the removal of water induces a modification of the local structure of almost every residue (Fig. 3j), although the average unit height decreases only by 1.3% (Fig. 3h). This result is in excellent agreement with the helix pitch changes obtained from our X-ray data.

Interestingly, the model predicts that local unit height changes are heterogeneous (Fig. 3j): residues in the overlap regions undergo mostly elongation deformation, whereas those in the gap shrink. Moreover, the calculations show a more homogeneous distribution of unit heights in the hydrated condition, which is in agreement with experimental results^29^. The model also reveals that unit height collapse in the gap region (identified by the segments with a unit height less than 0.45 nm in the dry conditions) occurs only in specific, mostly charged, segments of the collagen molecule (see Supplementary Fig. 6), suggesting that water plays an important role in preserving the triple-helical structure of these segments and screening interactions between side-chain charges.

### Significance for physiologically relevant conditions

Finally, the resulting shortening of the collagen is able to generate remarkable stresses even at high RH (>95%) corresponding to osmotic pressure changes, which are sufficiently low that they might occur even inside a fully hydrated environment. Indeed, the relation between tensile and osmotic stress close to origin (see inset of Supplementary Fig. 2d) shows that for a typical osmotic pressure of 0.4 MPa occurring in extracellular matrix^32^, the resulting tensile stress on collagen, about 0.25 MPa if calculated on the basis of the fibre dry area or about 0.15 MPa if the swelling of the collagen fibre is taken into account, should be in the order of peak muscle stresses.

**Figure 4:**
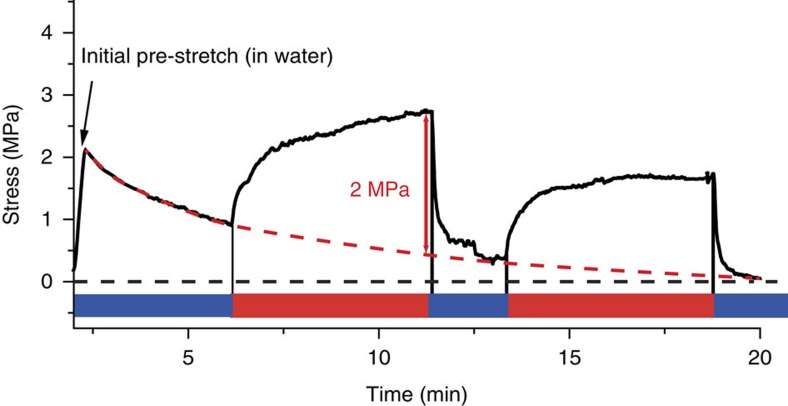
Osmotically induced force generation in condition similar to physiology. Stress generation in fibrolamellar cortical bone induced by cyclic changes of osmotic pressure (polyethylenglycol, 8,000 molecular weight, 50% wt exercises an osmotic pressure of about 9 MPa).

Unfortunately, the structural changes occurring with such small loads could not have been revealed by our methodology, which is why we chose to work with larger dehydrations that can only be achieved by adjusting the relative water vapour pressure around the collagen fibre. However, applying osmotic pressure through other means should give similar effects^33^. To support these considerations, we performed *in vitro* experiments where fibrolamellar cortical bone samples were cyclically subjected to osmotic pressure changes in completely hydrated conditions using polyethylene glycol typically used in osmotic stress experiments. Results are reported in Fig. 4 and demonstrate that even in the fully hydrated state, osmotic pressure induces tensile force in (mineralized) collagen.

## Discussion

From the experiments and from the model calculations, we conclude that water plays a crucial role in stabilizing the structure of the collagen molecule and is an essential and active part of the protein unit. The observations by *in situ* X-ray and Raman scattering are consistent with the full atomistic MD and lead to the following features of the drying process. First, the molecule and the fibril shrink by different amounts, 1.3% and 2.5%, respectively. Second, the dehydration is accompanied by a reduction of the gap/overlap ratio of the collagen fibrils. Third, the shrinkage of the triple-helix is inhomogeneous, as shown by the distribution of helix pitches and confirmed by MD simulations. Finally, Raman spectroscopy indicates conformational changes of the backbone upon drying.

**Figure 5:**
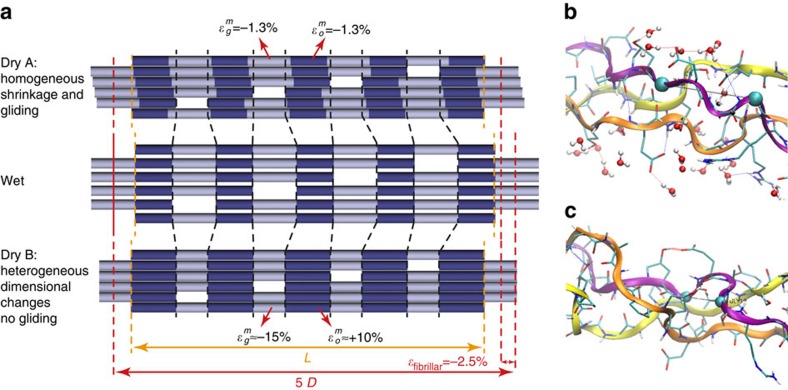
Models reflecting the X-ray scattering observations. (**a**) The top and the bottom drawings show two deformations of the fibril after drying without applied stress, both compatible with our results. The molecular arrangement in the hydrated fibril is shown in the centre. Molecular segments in the overlap region of the fibril are depicted in dark. The length of the triple-helix is *L* and the axial staggering period (including gap and overlap) is *D*. In both drying models, *L* decreases by 1.3%, *D* decreases by 2.5% and the gap region decreases relative to the overlap (dashed lines). In the first model (Dry A), we assume that the triple-helix shrinks homogeneously (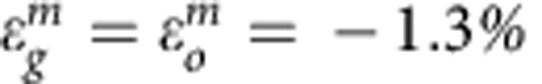) with a side-by-side gliding of neighbouring molecules, resulting in a relative shift of the dark segments. In the second model (Dry B), there is no side-by-side gliding but the molecule changes length inhomogeneously with an increase in the overlap (
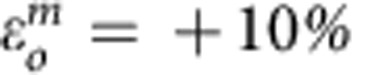
) and a shortening in the gap (
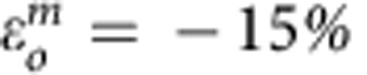
). Panels **b** and **c** show examples of collagen molecular structure extracted from the collagen microfibril MD model in wet (**b**) and dry (**c**) conditions, respectively.

These observations are translated into a simple model as shown in Fig. 5a. A *priori*, two possibilities exist that would explain the first two observations mentioned above. Indeed, a homogeneous shrinkage of the triple helix accompanied by a side-by-side gliding can reproduce all measured length changes as well as the change in gap/overlap ratio (Fig. 5a-DRY A). This type of structural change would just be the inverse of the deformation found by uniaxial stretching of fully hydrated collagen fibrils^14^,^15^,^34^,^35^. During passive stretching experiments, side-by-side gliding of neighbouring molecules was found at higher loads after the removal of an initial kinking of the molecules^15^. Our analysis of MD modelling results on drying collagen^22^ provides the insight that the triple-helix shrinks substantially in the gap region while it even extends in the overlap when water is removed (third observation). To illustrate the conformational changes, two snapshots of the collagen molecular structure in the gap region extracted from the full atomistic microfibril model in wet and dry conditions are shown in Fig. 5b,c, respectively. These results led us to propose a different model (Fig. 5a-DRY B) for contraction induced by dehydration than for extension under an applied force. Instead of a side-by-side gliding of the molecules, the change in gap/overlap ratio is rather achieved by shrinkage of the triple-helical molecules in the gap region, partially compensated by an expansion in the overlap region. This removes the need for the molecules to glide alongside each other to achieve the observed changes in gap/overlap ratio. In the case of dry tendons, water that may serve as ‘lubricant’ for a side-by-side gliding is actually removed so that is satisfying to see that the gap/overlap ratio change has another explanation. Some of the results of MD modelling hint towards the fact that, in reality, there could be a compromise between the two models with (a very small) residual side-by-side gliding accompanying the inhomogeneous shrinkage.

These molecular and supramolecular changes are responsible for the generation of very large stresses (up to 100 MPa) when the collagen undergoes a complete dehydration. However, our results indicate that also smaller changes in the osmotic pressure surrounding the collagen molecules (in the range of typical osmotic pressures exerted by proteoglycans, that is, 0.4 MPa) should produce macroscopic stresses comparable with peak muscle stresses. Whether this has a physiological importance in tendon, bone or other connective tissue is currently unknown, but the presence of proteoglycans typically associated with collagen in most extracellular tissues suggests that collagen is subjected to osmotic pressures in physiological conditions. Moreover, it is well-documented that collagen also contracts by the interaction with ions to the point that one can build machines based on this effect^36^,^37^. This ionic interaction is, however, likely to be even more complex at the molecular level than the simple dehydration which is of interest here. Moreover, the fact that the collagen staggering period in bone corresponds to the dry state of collagen^38^ strongly supports the idea that this mechanism could be operational in creating compressive residual stresses^39^,^40^ onto the mineral phase. Indeed, drying induces enormous tensile stress in collagen, up to 100 MPa, close to the strength of bone and tendon. Much like steel fibres in armoured concrete, collagen that shrinks axially during mineralization would put compressive load on the rest of the structure, protecting the mineral phase from tensile loads. All in all, these considerations suggest a potential and still unexplored active function of collagen fibrils, rather than a purely passive elastic behaviour.

## Methods

### Rat tail tendons

Fascicles of approximately 20 mm in length and 200 μm in thickness were dissected from the proximal end of the tail of Sprague–Dawley female rats aged 12 months. Animals were euthanized in deep anaesthesia by intracardial injection of 1 ml Rompun. The animal welfare as well as method of euthanasia was approved by the local authority Landesamt für Gesundheit und Soziales (Berlin, Germany).

### Mechanical testing

Samples were tested in a sealed chamber of volume of about 140 cm^3^. The chamber was kept at a constant temperature of 23° or 25° by means of cooling bath circulation thermostat (Huber). The humidity inside the chamber was controlled by means of ‘Wetsys (Setaram)’ humidity generator, which was working with a flow of 200 ml min^−1^. Temperature and humidity were monitored via a SHT75 digital humidity and temperature sensor (Sensirion) that was placed in the vicinity of the sample. Samples were clamped to two aluminium holders. The strain was controlled by one of the holders that connects to a PI (Physik-Instrumente) M-404 linear motor stage (resolution of 2 μm), whereas the axial tensile force was measured using a Honeywell R-30 load cell (50 N max. load), attached to the other holder.

The standard deviation of the measured force background noise over more than 60,000 points was 7.5 mN. The force changes associated with step changes in humidity were always more than three times standard deviation of the force background noise.

The mechanical tests have been performed in zero-stress and successively in iso-strain mode measurements for each sample. In a typical test, the length change (zero-stress) or the arising force (iso-strain) were measured, whereas the RH in the chamber was changed stepwise. For all measurements, a wet sample was clamped initially at a length of about 10–15 mm and equilibrated for at least 30 min in wet conditions, driving the motors to keep a constant force of about 50 mN (which is less than 2–3% of the typical highest forces reached after completely drying out the sample at fixed length). After the equilibrium wet length *L*_0_ was reached, the motors were stopped and the arising force was measured during stepwise changes of RH. In contrary, in zero-stress experiments, the motors continued driving keeping the low force (50 mN) constant while the humidity was changed. At humidity higher than 20% RH, for each step, a 2-h time was allowed for equilibration, although the environmental equilibrium of the chamber was reached already after 30 min. At lower humidity, the time for sample equilibrium at each step was increased to 3–5 h.

Custom written software was developed in house to continuously monitor distances on calibrated optical images (precision on the length determination was about 1 μm). With this, in both zero-stress and iso-strain experiments, the changes in the fibre diameter, as well as the axial strains, could be determined by means of video extensometry. For the strain determination [*ε*_L_=(*L*−*L*_0_)/*L*_0_], the length *L*_0_ and thickness in wet condition were taken, whereas for the stress, the fibre size in dry state was used. The precise areas of the irregular fibre cross-sections were measured by means of μ-computed tomography (CT). CT data were acquired rotating the samples of 180° every 2° and using 1 s as acquisition time and the image pixel size (4.5 μm per pixel) was calibrated using a standard. For the force per molecule calculation, the macroscopic stress was multiplied by the area of a dry molecule 1.0479, nm^2^ per molecule, which results from the assumption of a hexagonal equidistant arrangement of molecules in a distance of 1.1 nm (1.55 for wet condition). For the assessment of the molecular stress, we repeated the force measurement in iso-strain conditions 12 times, measuring the area of the fascicle with CT every time, and the values we report are the average value with the standard deviation of the calculated stress distribution.

### Raman spectroscopy

For Raman microspectroscopy, a continuous laser beam was focused down to a micrometre-sized spot on the sample through a confocal Raman microscope (CRM200, WITec) equipped with a piezo-scanner (P-500, Physik Instrumente). A diode-pumped 785-nm near-infrared laser excitation (Toptica Photonics AG) was used in combination with a × 20 (Nikon, numerical aperture=0.4) microscope objective. The spectra were acquired using a CCD (PI-MAX, Princeton Instruments Inc.) behind a grating (300 g mm^−1^) spectrograph (Acton, Princeton Instruments Inc.) with a spectral resolution of ~6 cm^−1^. Thirty accumulations with integration time of 1 s were used for each single spectrum. The ScanCtrlSpectroscopyPlus (version 1.38, WITec) and WitecProjectPlus (version 2.02, WITec) were used for the experimental setup and spectral data processing, respectively.

### X-ray diffraction data

SAXS measurements applying synchrotron radiation were performed at the μspot beamline, BESSY II, at the Helmholtz Zentrum Berlin. X-ray patterns were recorded with a 2D CCD detector (MarMosaic 225, Rayonix Inc. with a pixel size of 73 μm and an array of 3,072 × 3,072 pixels. For the acquisition of the 2D pattern, the energy of the X-ray beam (100 μm in diameter) of 15 keV and a sample-to- detector distance of 652.3 mm were calibrated using a silver behenate (AgBeh) standard. In order to reach the small momentum transfer needed to reach the first three meridional peaks, a smaller beam size of 30 μm was used, an energy of 8 keV and a sample-to-detector distance of 851.9 mm were calibrate with the same standard. In both cases, short acquisition times were used to collect the data in order to avoid possible radiation damage^41^. Per every experimental condition, three 2D patterns of 60 s each from three different regions (separated from each other 100 μm in z) were acquired and the same region was never used twice to acquire a diffraction pattern. A force raise/decrease as a consequence of the shining of the sample with the beam was never observed. For detailed results see Supplementary Figs 3 and 4.

All patterns were corrected for empty beam background and variations in incident beam intensity. For the evaluation of the first three meridional peaks, the 2D SAXS patterns were integrated in the equatorial direction using the Projection function of Fit2D software^42^. The one-dimensional intensities *I*(*q*_mer_) of meridional peaks were fitted by a Lorenzian curve to find the peak positions *q*_mer_ for the modulus of the scattering wave vector and its integrated intensity. The staggering period *D* under different hydration conditions was calculated fitting the intensities of the 20th reflection.

### Collagen microfibril MD model

The collagen microfibril model is generated based on the *in situ* structure of full-length collagen type I molecule^43^ (Protein Data Bank identification code 3HR2), which has a triclinic unit cell (*a*≈ 40.0 Å, *b*≈27.0 Å, *c*≈678 Å, *α*≈89.2°, *β*≈94.6°, *γ*≈105.6°). The microfibril model includes the N-, C-telopeptide domains and the full type I collagen sequences in each chain. For further information, we refer the reader to a previous paper with details on the *in situ* structure of collagen and on the development of the full atomistic collagen microfibril model^22^.

Full atomistic simulations are carried out using the GROMACS 4.0 code with the GROMOS 43a1 force field. An energy minimization is performed by a steepest descent algorithm followed by *NPT* MD simulations at temperature of 310 K and with 1 bar pressure. The mechanical properties of the collagen microfibril model have been tested and shown to be in agreement with experimental measurements as reported in ref. 22. Each chain of a collagen molecule is a repeating primary sequence of (Gly-X-Y)_*n*_ and the three chains are staggered with respect to each other (Fig. 3a). The positions of the repeated Gly therefore capture the characteristic structures of collagen molecules. We use the C_α_ atom of each Gly residue as a measurement point to characterize molecular structure. The centre of mass of C_α_ atoms of the *i*-th Gly residue in three chains is computed and the unit height is measured by the distance between adjacent centre of masses. More information on the analysis method has been reported in ref. 44.

## Author contributions

A.M., L.B. and P.F. conceived the idea and wrote the manuscript. R.S., H.M., A.M. and L.B. carried out the experiments and evaluated the data. S.-W.C. and M.J.B. performed computer simulations. All authors contributed to the interpretation of the results and commented on the manuscript.

## Additional information

**How to cite this article:** Masic, A. *et al*. Osmotic pressure induced tensile forces in tendon collagen. *Nat. Commun.* 6:5942 doi: 10.1038/ncomms6942 (2015).

## Supplementary Material

Supplementary Information Supplementary Figures 1-6, Supplementary Methods, and Supplementary References
