# Supplementary material for: Osmotic pressure induced tensile forces in tendon collagen
Source: Nat Commun. 2015 Jan 22;6:5942. doi: 10.1038/ncomms6942 (PMC4354200; doi:10.1038/ncomms6942)
Supplement: Supplementary Information — Supplementary Figures 1-6, Supplementary Methods, and Supplementary References [file ncomms6942-s1.pdf]

Supplementary figures

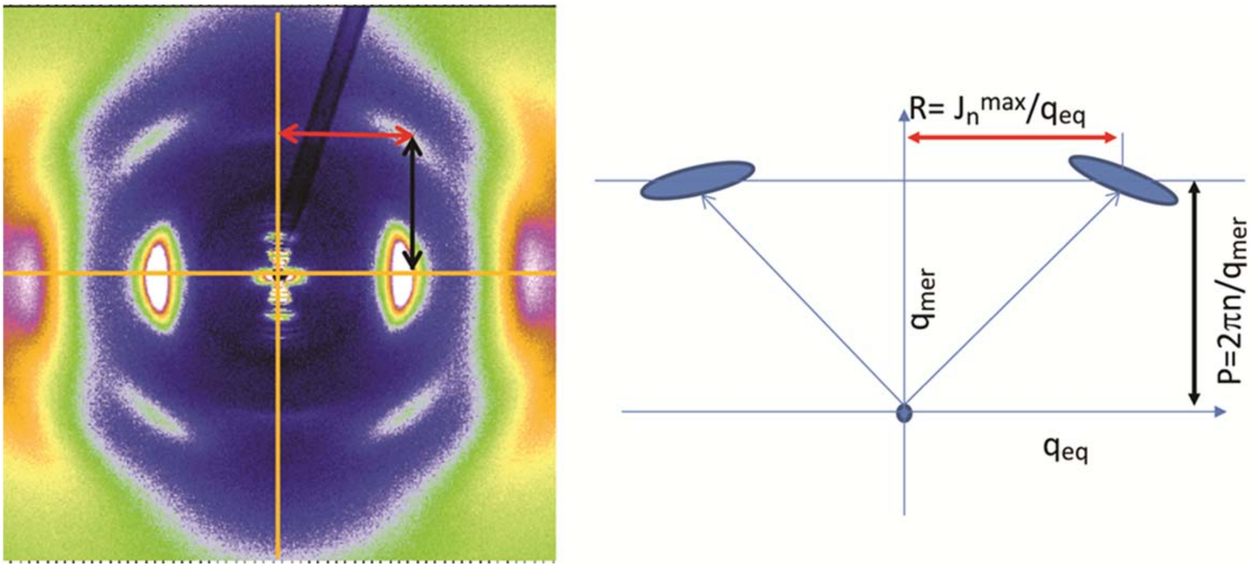

Supplementary Figure 1: A typical 2D x-ray diffraction pattern of RTT. The position of helix reflection peaks (Q space) is converted into the triple-helix diameter and pitch (real space) as described in the main text.

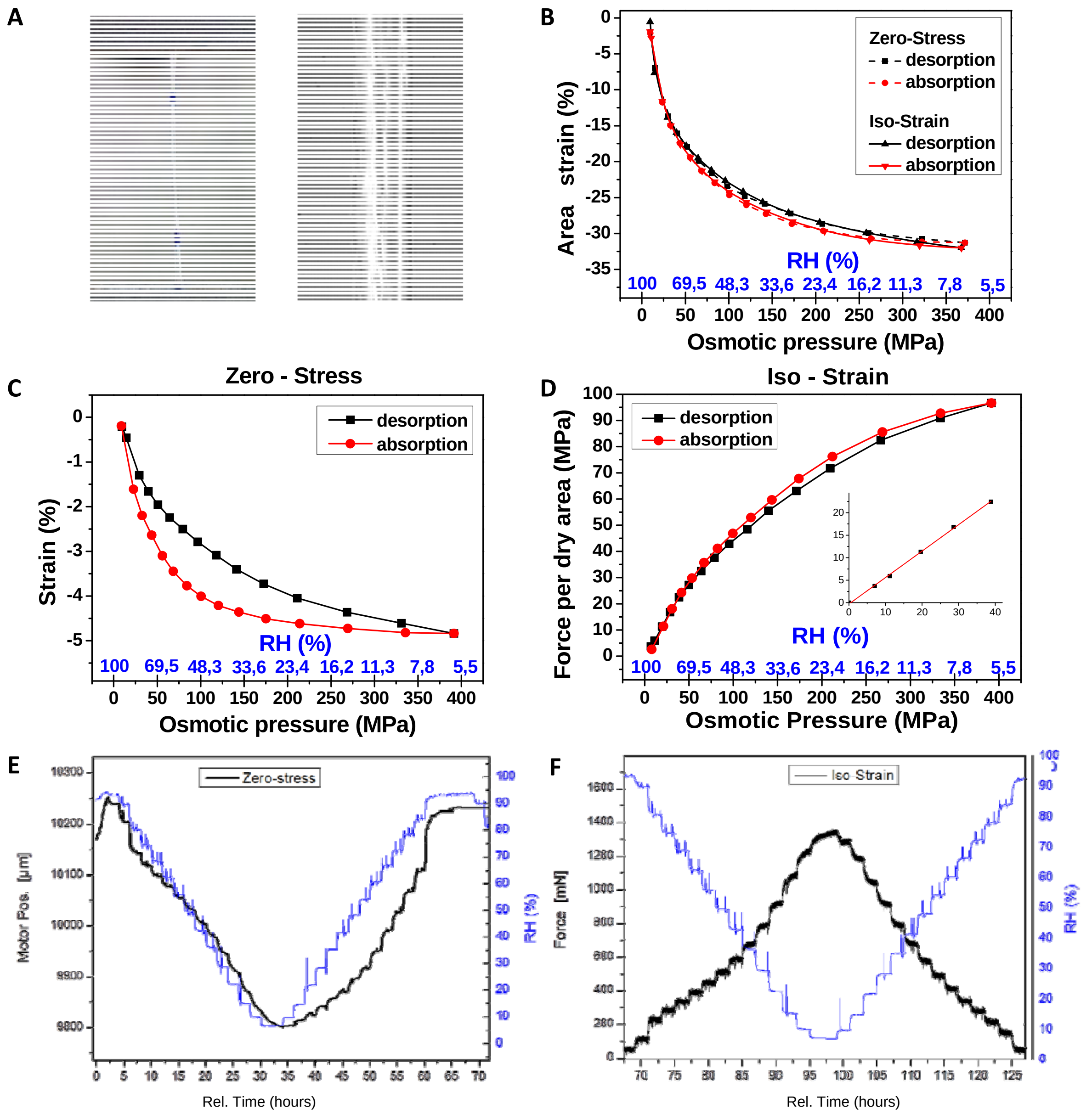

Supplementary Figure 2: Zero-stress and iso-strain macroscopic video extensometric experiments of RTT in length and width during humidity changes. (A) Optical camera view of the clamped sample during the experiment. (B) Cross-section area of the fibres is measured and plotted in function of RH for both zero-stress and iso-strain experiment. In both experiments the fibre area shrinks around 30% upon drying. The area strain was calculated from the measured diameter change assuming that the fibre has a perfect cylindrical shape. The x-axis is plotted in both relative humidity and osmotic pressure units. (C) In relation to the longitudinal direction in the case of zero-stress experiment the shrinking of the fibrefibre is observed and reaches about 5% of total strain in length at 5% RH. (D) On the other hand, in the iso-strain experiment where fibre is unable to shrink, the internal stresses are generated and reach values between 90 and 120 MPa at 5% RH. At high relative humidity (inset), between fully wet and 70%, the tensile stress generated with drying (σ) is linear with the osmotic pressure change (Π) and can be expressed as $\sigma = (0.6 \pm 0.03)*\Pi$ (red line in inset). (E) and (F) Time dependent length changes (E, black curve) and force generation (F, black curve) induced by RH (blue curves).

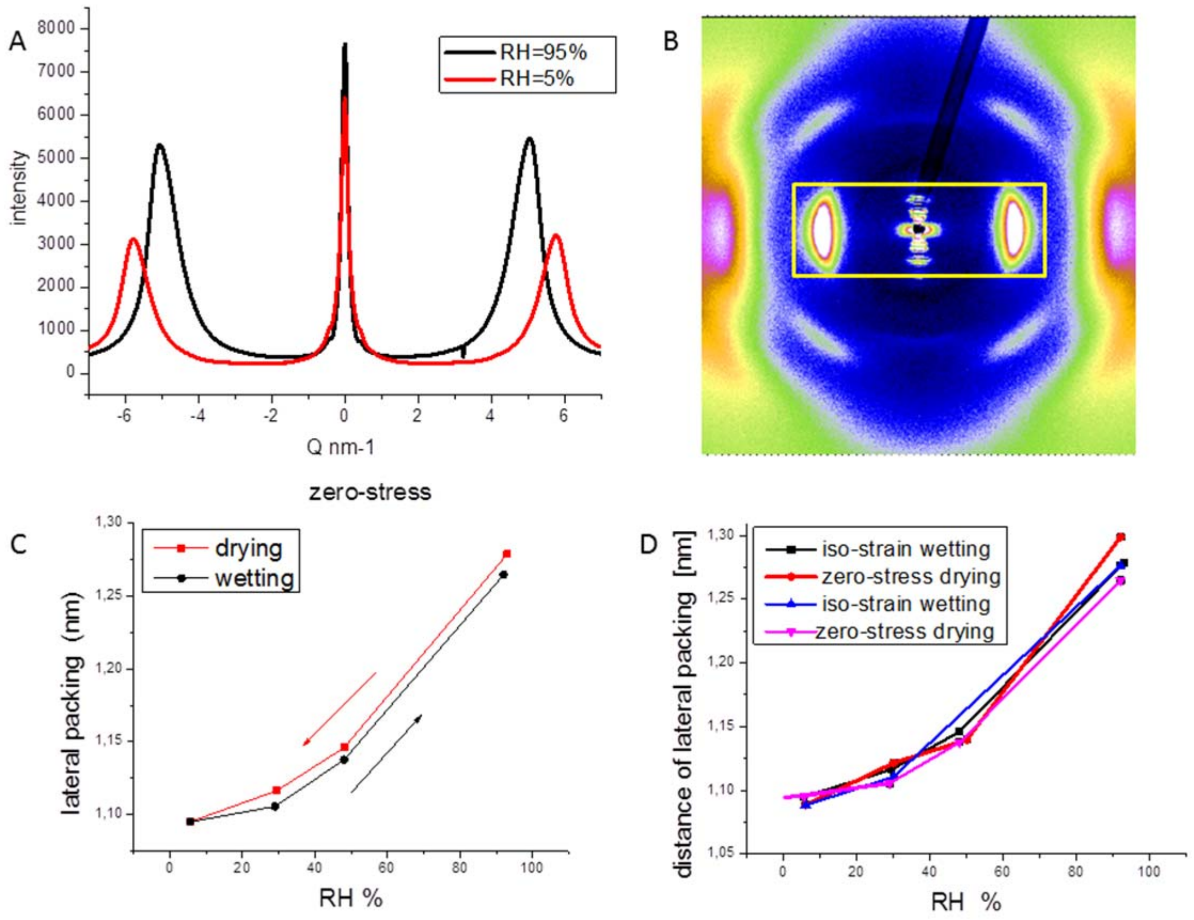

Supplementary Figure 3: *In situ* x-ray lateral analysis of RTT as a function of humidity during iso-strain and zero-stress experiments. The 2D pattern (B) is evaluated using the "Projection" function of FIT2D in lateral direction (yellow box). The shift in momentum transfer Q (A) is converted into the lateral packing distance as a function of relative humidity (C). No change in the lateral packing is found for iso-strain and zero-stress conditions during wetting or drying (D).

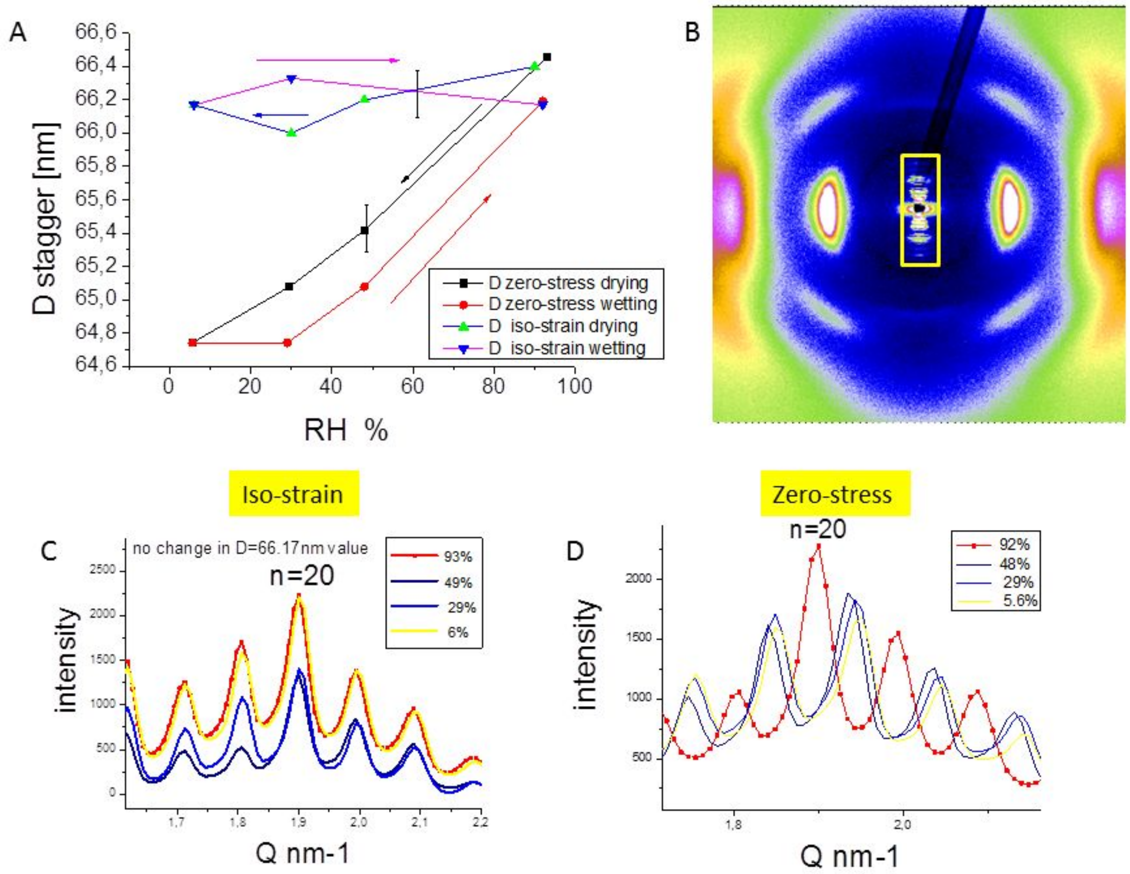

Supplementary Figure 4: *In situ* x-ray axial staggering analysis of RTT as a function of humidity during iso-strain and zero-stress experiments. The 2D pattern (B) is evaluated using the "Projection" function of FIT2D in axial direction (yellow box). As a function of RH, under iso-strain conditions the peak positions in Q space remain unchanged (C), while they shift under zero-stress condition (D). The corresponding staggering distance D is shown in (A) for all measurements.

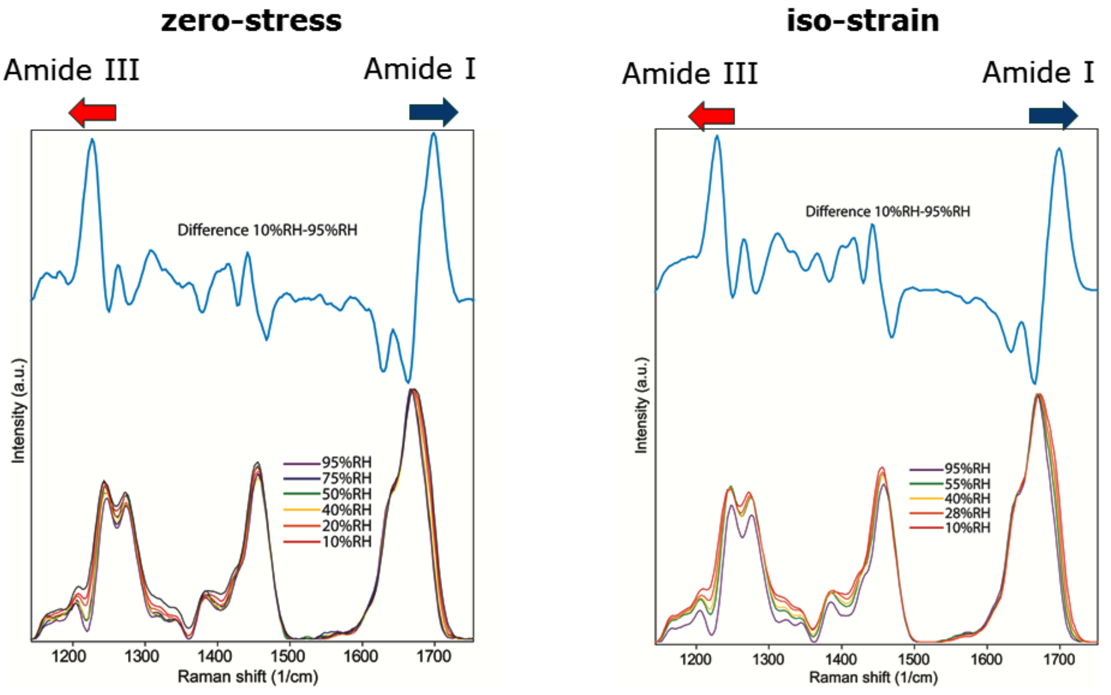

Supplementary Figure 5: *In situ* Raman spectroscopy of RTT during drying. Series of spectra acquired at different RH for zero-stress (left) and iso-strain (right) experiment. Blue lines in both datasets are difference spectra between RH=10% and RH=95% aiming to highlight the main differences in spectral features. The changes in both zero-stress and iso-strain are consistent with alterations in hydrogen bonding observed in the amide I (1600–1700 cm^{-1}, predominantly C=O stretching) and amide III (1200–1300 cm^{-1}, mostly NH in-plane bending and CN stretching) modes. In particular, as RH decreases, the more intense component in the amide I band displays an upshift in frequency from 1665 to 1672 cm^{-1} along with a decrease in the position of the low frequency component of Amide III at 1245 cm^{-1}. These observations are consistent with an increase in hydrogen bonding strength as RH increases. It is well established that when hydrogen bonding involves one of the atoms in a stretching vibration, a frequency decrease is observed whereas the opposite occurs for a bending mode.

alpha-1 chain:

GPMGPSGPRGLPGPPGAPGPQGFQGPPGEPGEPGASGPMGPRGPPGPPGKNGDDGEAGKPGR
PGERGPPGPQGARGLPGTAGLPGMKGHRGFSGLDGAKGDAGPAGPKGEPGSPGENGAPGQMG
PRGLPGERGRPGAPGPAGARGNDGATGAAGPPGPTGPAGPPGFPGAVGAKGEAGPQGPRGSE
GPQGVRGEPGPPGPAGAAGPAGNPGADGQPGAKGANGAPGIAGAPGFPGARGPSGPQGPGGP
PGPKGNSGEPGAPGSKGDTGAKGEPGPVGVQGPPGPAGEEGKRGARGEPGPTGLPGPPGERG
GPGSRGFPGADGVAGPKGPAGERGSPGPAGPKGSPGEAGRPGEAGLPGAKGLTGSPGSPGPD
GKTGPPGPAGQDGRPGPPGPPGARGQAGVMGFPGPKGAAGEPGKAGERGVPGPPGAVGPAGK
DGEAGAQGPPGPAGPAGERGEQGPAGSPGFQGLPGPAGPPGEAGKPGEQGVPGDLGAPGPSG
ARGERGFPGERGVQGPPGPAGPRGANGAPGNDGAKGDAGAPGAPGSQGAPGLQGMPGERGAA
GLPGPKGDRGDAGPKGADGSPGKDGVRGLTGPIGPPGPAGAPGDKGESGPSGPAGPTGARGA
PGDRGEPGPPGPAGFAGPPGADGQPGAKGEPGDAGAKGDAGPPGPAGPAGPPGPIGNVGAPG
AKGARGSAGPPGATGFPGAAGRVGPPGPSGNAGPPGPPGPAGKEGGKGPRGETGPAGRPGEV
GPPGPPGPAGEKGSPGADGPAGAPGTPGPQGIAGQRGVVGLPGQRGERGFPGLPGPSGEPGK
QGPSGASGERGPPGPMGPPGLAGPPGESGREGAPGAEGSPGRDGSPGAKGDRGETGPAGPPG
APGAPGAPGPVGPAGKSGDRGETGPAGPAGPVGPVGARGPAGPQGPRGDKGETGEQGDRGIK
GHRGFSGLQGPPGPPGSPGEQGPSGASGPAGPRGPPGSAGAPGKDGLNGLPGPIGPPGPRGR
TGDAGPVGPPGPPGPPGPPGPP

alpha-2 chain:

GPMGLMGPRGPPGAAGAPGPQGFQGPAGEPGEPGQTGPAGARGPAGPPGKAGEDGHPGKPGR
PGERGVVGPQGARGFPGTPGLPGFKGIRGHNGLDGLKGQPGAPGVKGEPGAPGENGTPGQTG
ARGLPGERGRVGAPGPAGARGSDGSVGPVGPAGPIGSAGPPGFPGAPGPKGEIGAVGNAGPA
GPAGPRGEVGLPGLSGPVGPPGNPGANGLTGAKGAAGLPGVAGAPGLPGPRGIPGPVGAAGA
TGARGLVGEPGPAGSKGESGNKGEPGSAGPQGPPGPSGEEGKRGPNGEAGSAGPPGPPGLRG
SPGSRGLPGADGRAGVMGPPGSRGASGPAGVRGPNGDAGRPGEPGLMGPRGLPGSPGNIGPA
GKEGPVGLPGIDGRPGPIGPAGARGEPGNIGFPGPKGPTGDPGKNGDKGHAGLAGARGAPGP
DGNNGAQGPPGPQGVQGGKGEQGPPGPPGFQGLPGPSGPAGEVGKPGERGLHGEFGLPGPAG
PRGERGPPGESGAAGPTGPIGSRGPSGPPGPDGNKGEPGVVGAVGTAGPSGPSGLPGERGAA
GIPGGKGEKGEPGLRGEIGNPGRDGARGAPGAVGAPGPAGATGDRGEAGAAGPAGPAGPRGS
PGERGEVGPAGPNGFAGPAGAAGQPGAKGERGAKGPKGENGVVGPTGPVGAAGPAGPNGPPG
PAGSRGDGGPPGMTGFPGAAGRTGPPGPSGISGPPGPPGPAGKEGLRGPRGDQGPVGRTGEV
GAVGPPGFAGEKGPSGEAGTAGPPGTPGPQGLLGAPGILGLPGSRGERGLPGVAGAVGEPGP
LGIAGPPGARGPPGAVGSPGVNGAPGEAGRDGNPGNDGPPGRDGQPGHKGERGYPGNIGPVG
AAGAPGPHGPVGPAGKHGNRGETGPSGPVGPAGAVGPRGPSGPQGIRGDKGEPGEKGPRGLP
GLKGHNGLQGLPGIAGHHGDQGAPGSVGPAGPRGPAGPSGPAGKDGRTGHPGTVGPAGIRGP
QGHQGPAGPPGPPGPPGPPGVS

Supplementary Figure 6: Segments of the type I collagen molecule primary amino acid sequence. The local conformation of the type I collagen molecule is highly dependent on its sequences. The sequences of the six segments undergoing significant collapse, which are identified by the segments with a unit height less than 0.45 nm in the dry condition, are highlighted.

Supplementary Methods

Further details on data collection

The data presented in this work were acquired from tendons extracted from 3 different rat tails. We measured the force raise and the length strain associated with the full range of humidity changes (without x-rays) 4 times and the plots in Fig. 2 (green and black respectively for iso-strain and zero-stress) represent the average curves obtained from these datasets. For the error bars, the standard deviation of the data at every point was taken. For the assessment of the molecular stress, we repeated the force measurement in iso-strain conditions from wet to completely dry (without equilibrating at intermediate RHs) 12 times, measuring the area of the fascicle with CT every time, and the values we report (107pN molecule^{-1} ± 16 pN molecule^{-1}) are the average value and the standard deviation of the calculated stress distribution. The *in situ* x-ray experiments were repeated on 2 different samples (both for zero-stress and iso-strain) and the reported graphs represent the average curve obtained averaging the two datasets. The error bars represent the maximum variation of the data observed among all the experimental conditions.

Further analysis of X-ray data

To extract the helix parameters (*i.e.* pitch and radius) from the SAXS measurements, following the conclusions of Okuyama[1], the 2D diffraction patterns presented in Fig. 2 in the q space were assigned to the collagen structural parameter space using the 7/2 model for the collagen triple helix structure{Okuyama, 2008 #2389}. For every pixel with coordinates (q_{eq}, q_{mer}), it holds (see also Supplementary Fig. 1):

$$P = \frac{2\pi n}{q_{\mathrm{mer}}} \qquad (1)$$

and

$$R = \frac{J_n^{\mathrm{max}}}{q_{\mathrm{eq}}} \qquad (2)$$

where P is the axial period of the collagen molecule, that correspond to 1/3 of the pitch length, n is the order of the layer line and J_n^{max} is the position of the first maximum of the nth-order Bessel function of the first kind.

As we considered the 7/2 model for collagen, the reflection studied here is the one corresponding to the second layer line, so that n=2 and J_n^{max} is 3.

The 1D distribution of helix pitches and radii were obtained projecting the aforementioned 2D maps on the vertical and horizontal axes, respectively. The average values were obtained integrating over the distribution function after having removed the background.

Model of collagen drying

The values of the strain presented in Fig. 4a were obtained considering the following: in wet conditions, the staggering period (the sum of gap (G) and overlap (O)) is D= G+O = 66 nm, as measured by x-rays. Also, the gap to overlap ratio is known to be 0.54:0.46, so that the value of G and O can be readily calculated[2,3]. After dehydration, the D period shrinks of about 2.5% during zero-stress measurements, while the molecular length L only by about 1.3%. If a homogeneous shrinkage over all the collagen molecule is assumed, i.e. $\varepsilon_G^{\mathrm{m}} = \Delta G/G$and $\varepsilon_{\mathrm{O}}^{\mathrm{m}} = \Delta O/O$ are both -1.3%, this would mean that, over 5 periods (330 nm), the collagen molecules should glide side by side of the residual 1.2%, i.e. of about 4 nm.

On the other hand, the same molecular and fibrillar strains can be attained considering that no interfibrillar gliding occurs, but the gap and overlap regions undergo different length changes upon drying. In this case the relations that have to be satisfied are:

$$O^{\mathrm{d}} + G^{\mathrm{d}} = D^{\mathrm{w}}(1 - 0.025) \qquad (3)$$

and

$$5O^{\mathrm{d}} + 4G^{\mathrm{d}} = L^{\mathrm{w}}(1 - 0.013) \qquad (4)$$

where the superscripts d and w indicate respectively the dry and the wet conditions. This set of equations is easily solved giving:

$$O^{\mathrm{d}} = L^{\mathrm{w}}(1 - 0.013) - 4D^{\mathrm{w}}(1 - 0.025) \qquad (5)$$

and

$$G^{\mathrm{d}} = 5D^{\mathrm{w}}(1 - 0.025) - L^{\mathrm{w}}(1 - 0.013) \qquad (6)$$

The strains calculated from these equations are about -12% ± 5% (approximated to 15% in the Fig. 4A and +9% ± 5% (approximated to 10 in the Fig. 4A) respectively for the gap and the overlap regions.

The gap to overlap ratio can also be evaluated through the intensities of the first orders peaks related to the staggering period following the indications of Sasaki et al.[4]

Supplementary References

1. Okuyama, K. Revisiting the Molecular Structure of Collagen. *Conn. Tissue Res.* **49**, 299-310 (2008).

2. Schmitt, F. O., Hall, C. E. & Jakus, M. A. Electron microscope investigations of the structure of collagen. *J. Cell. Comp. Physiol.* **20**, 11-33 (1942).

3. Orgel, J. P., Wess, T.J. & Miller, A. *Structure* **8**, 137-142 (2000).

4. Sasaki, N., Shukunami, N., Matsushima, N., & Izumi, Y. Time-resolved X-ray diffraction from tendon collagen during creep using synchrotron radiation. *J. Biomech.* **32**, 285-292 (1999).
